# A commentary on ‘Effectiveness of artificial intelligence-assisted colonoscopy in early diagnosis of colorectal cancer: a systematic review’

**DOI:** 10.1097/JS9.0000000000000598

**Published:** 2023-07-18

**Authors:** Si-Un Frank Chiu, Kuo-Chuan Hung, Chong-Chi Chiu

**Affiliations:** a Department of Computer Science; b Department of Economics, Brown University, Providence, Rhode Island, USA; c Department of Anesthesiology, Chi Mei Medical Center; d Department of Hospital and Health Care Administration, College of Recreation and Health Management, Chia Nan University of Pharmacy and Science, Tainan; e Department of General Surgery; f Department of Medical Education and Research, E-Da Cancer Hospital; g School of Medicine, College of Medicine, I-Shou University, Kaohsiung, Taiwan

*Dear Editor*,

Screening colonoscopy could effectively lower colorectal cancer (CRC) risk and lead to a considerable financial burden. Artificial intelligence (AI), specifically computer-aided detection (CADe), can revolutionize endoscopy by improving the detection of adenomas and reducing the risk of post-colonoscopy CRC. This technology uses advanced computer algorithms to analyze colonoscopy images, help identify and confirm the presence of polyps and predict polyp histopathology accurately. By utilizing AI algorithms to analyze colonoscopy images, AI-assisted colonoscopy (AIC) is expected to overcome human limitations, such as fatigue and variability in interpretation, resulting in a more accurate and reliable detection process. In daily clinical practice, smaller lesions are often more challenging to identify during routine colonoscopy and can easily be missed by even experienced endoscopists^1^. Thus, the higher sensitivity of AIC in detecting smaller adenomas is particularly noteworthy. AIC’s ability to detect and characterize these subtle lesions could increase the chances of early intervention and subsequent favorable patient outcomes. Moreover, improved detection rates can reduce the need for repeated colonoscopies, minimize patient discomfort, and optimize healthcare resource allocation. Although AIC has shown promising results in Mehta’s systematic review^2^, two recent studies have raised questions about the actual benefits of these technologies.

Hassan *et al*.^3^ compared *in vivo* two prevalent computer-aided diagnoses (CADx) systems and found high diagnostic concordance between them, but unassisted endoscopic diagnosis outperformed CADx in terms of technical accuracy and clinical outcomes, indicating that the expertise of the endoscopists still plays a crucial role in accurate polyp characterization. This finding also suggests that the current CADx systems may still need to be prepared to replace conventional histopathologic analysis, particularly for more complex polyps.

Ladabaum *et al*.^4^ assessed the impact of CADe devices on colonoscopy quality metrics in six medical centers under the Stanford Colonoscopy Quality Assurance Program. However, they found no significant improvement in adenoma detection rate (ADR) or other detection rates, procedure times, or resection rates, suggesting that the actual benefits of CADe may be limited. These findings contrast with some randomized trials quoted by Mehta’s study^2^. However, factors such as overreliance on CADe notifications and the high baseline ADR of the endoscopists may have contributed to these outcomes.

While there has been significant enthusiasm about the potential of CADx and CADe systems to improve the accuracy and efficiency of colonoscopy procedures, the findings of these two studies suggest that the actual impact and effectiveness of AIC may not be as significant as initially anticipated. However, we recommend the need for cautious optimism when integrating AI-driven solutions into clinical practice. While the potential of AI in colonoscopy is undeniable, it is crucial to thoroughly evaluate the benefits, limitations, and potential risks associated with these technologies. Besides, the widespread dissemination and actual performance of CADe and CADx colonoscopy technology are essential to fully assess its impact and effectiveness. Thus, the success of AI implementation in gastroenterology will heavily rely on appropriate educational initiatives. Training programs and guidelines should address the technical aspects of colonoscopy skills and the optimal utilization of AI solutions. In other words, it is essential to ensure that gastroenterologists have the knowledge and expertise to effectively leverage AI technologies while maintaining their critical role in the diagnostic and therapeutic process.

Integrating AI technology in colonoscopy contributes to the standardization of procedures and the development of best practices. There is considerable variability in the quality and efficacy of colonoscopies performed by different practitioners^5^. AIC is expected to provide a consistent and objective approach to lesion identification and characterization, reducing operator dependency and ensuring higher accuracy across the board. This standardization not only improves the quality of individual procedures but also facilitates benchmarking and quality improvement initiatives at a broader level. However, we should consider the economic implications of integrating AIC into routine clinical practice. While some experts demand that AIC shows promise in improving detection rates and reducing long-term treatment costs, the initial investment in AI technology and the associated infrastructure can be substantial. It is essential to conduct cost-effectiveness studies to evaluate the long-term financial implications and determine the feasibility of widespread adoption, especially in developing countries.

## Ethical approval

This is only a commentary, not research involving patients. No ethical approval is required.

## Consent

This is only a commentary, not research involving patients. No patient consent is required.

## Sources of funding

This is a commentary. We have no funding for our commentary.

## Author contribution

S.-U.F.C.: conceptualization and writing the original draft; K.-C.H.: validation; C.-C.C.: conceptualization, supervision, submission, and correspondence.

## Conflicts of interest disclosure

There are no conflicts of interest in our commentary.

## Research registration unique identifying number (UIN)

This is a commentary to a study published in ‘International Journal of Surgery’. No UIN is required.

## Guarantor

Professor Chong-Chi Chiu.

## Data availability statement

This is only a commentary to a study published in ‘International Journal of Surgery’. There are no research data in our commentary.

## Provenance and peer review

This paper was not invited.

## References

[1] Lee J Park SW Kim YS. Risk factors of missed colorectal lesions after colonoscopy. Medicine (Baltimore) 2017;96:e7468. 28682916 10.1097/MD.0000000000007468 PMC5502189

[2] Mehta A Kumar H Yazji K. Effectiveness of artificial intelligence-assisted colonoscopy in early diagnosis of colorectal cancer: a systematic review. Int J Surg 2023;109:946–52. 36917126 10.1097/JS9.0000000000000285 PMC10389330

[3] Hassan C Sharma P Mori Y. Comparative performance of artificial intelligence optical diagnosis systems for leaving in situ colorectal polyps. Gastroenterology 2023;164:467–469. 36328079 10.1053/j.gastro.2022.10.021

[4] Ladabaum U Shepard J Weng Y. Computer-aided detection of polyps does not improve colonoscopist performance in a pragmatic implementation trial. Gastroenterology 2023;164:481–483. 36528131 10.1053/j.gastro.2022.12.004 PMC11521095

[5] Spada C Koulaouzidis A Hassan C. Colonoscopy quality across Europe: a report of the European Colonoscopy Quality Investigation (ECQI) Group. Endosc Int Open 2021;9:E1456–E1462. 34540535 10.1055/a-1486-6729 PMC8445680

